# Assessing and reporting heterogeneity in treatment effects in clinical trials: a proposal

**DOI:** 10.1186/1745-6215-11-85

**Published:** 2010-08-12

**Authors:** David M Kent, Peter M Rothwell, John PA Ioannidis, Doug G Altman, Rodney A Hayward

**Affiliations:** 1 Institute for Clinical Research and Health Policy Studies, Tufts Medical Center, Boston, MA, USA; 2 Department of Clinical Neurology, John Radcliffe Hospital, Oxford, UK; 3 Department of Hygiene and Epidemiology, University of Ioannina School of Medicine, Ioannina, Greece; 4 Centre for Statistics in Medicine, University of Oxford, Oxford, UK; 5 VA Ann Arbor Healthcare System & the Department of Internal Medicine, University of Michigan, Ann Arbor, MI, USA

## Abstract

Mounting evidence suggests that there is frequently considerable variation in the risk of the outcome of interest in clinical trial populations. These differences in risk will often cause clinically important heterogeneity in treatment effects (HTE) across the trial population, such that the balance between treatment risks and benefits may differ substantially between large identifiable patient subgroups; the "average" benefit observed in the summary result may even be non-representative of the treatment effect for a typical patient in the trial. Conventional subgroup analyses, which examine whether specific patient characteristics modify the effects of treatment, are usually unable to detect even large variations in treatment benefit (and harm) across risk groups because they do not account for the fact that patients have multiple characteristics simultaneously that affect the likelihood of treatment benefit. Based upon recent evidence on optimal statistical approaches to assessing HTE, we propose a framework that prioritizes the analysis and reporting of multivariate risk-based HTE and suggests that other subgroup analyses should be explicitly labeled either as primary subgroup analyses (well-motivated by prior evidence and intended to produce clinically actionable results) or secondary (exploratory) subgroup analyses (performed to inform future research). A standardized and transparent approach to HTE assessment and reporting could substantially improve clinical trial utility and interpretability.

## Introduction

When the Scottish epidemiologist Archie Cochrane suggested that clinical practice should principally be guided by rigorously designed evaluations, in particular randomized clinical trials (RCTs), the reaction of the medical profession was largely negative. Critics suggested that relying on impersonal statistically-derived "evidence" based on averages to determine clinical decision-making was antithetical to the practice of medicine, which should rather be based on a physician's expertise, acumen and clinical experience, and on knowing the individual patient and considering what is best for each person given their individual circumstances and needs [1-3].

Although "evidence-based medicine" has become the dominant paradigm for shaping clinical recommendations and guidelines, recent work demonstrates that many clinicians' initial concerns about "evidence-based medicine" come from the very real incongruence between the overall effects of a treatment in a study population (the summary result of a clinical trial) and deciding what treatment is best for an individual patient given their specific condition, needs and desires (the task of the good clinician) [4-7]. The answer, however, is *not *to accept clinician or expert opinion as a replacement for scientific evidence for estimating a treatment's efficacy and safety, but to better understand how the effectiveness and safety of a treatment varies across the patient population (referred to as heterogeneity of treatment effect [HTE]) so as to make optimal decisions for each patient.

The conventional method of examining whether treatment effects vary in a trial population is to divide patients into subgroups based on potentially influential characteristics. The main problem with the conventional approach is that there are too many characteristics that can potentially influence treatment effect. This leads to myriad subgroup analyses which are typically both underpowered and vulnerable to spurious false positive results due to multiple comparisons. For these reasons, subgroup analyses are usually "exploratory" and rarely actionable, leaving the clinician to assume that all patients meeting trial inclusion criteria should be similarly treated.

Herein, we propose a framework that directly addresses the problem of multiplicity in two ways. First, our framework prioritizes the analysis and reporting of multivariate risk-based HTE, over conventional "one-variable-at-a-time" subgroup analysis. This recommendation is based on an understanding that HTE emerges from just a few fundamental risk dimensions. These dimensions--which include the risk of the primary study outcome (the main focus of our proposed approach), competing risk, the risk of treatment-related harm and direct treatment-effect modification [5-8]--can often be summarized using multivariate prediction models, greatly simplifying subgroup analyses and substantially improving statistical power[9]. Second, this framework proposes that other subgroup analyses should be explicitly labeled either as primary subgroup analyses (well-motivated by prior evidence and intended to produce clinically actionable results), which should be few in number and appropriately adjusted for multiple comparisons, or secondary (exploratory) subgroup analyses (performed to inform future research).

### Why the overall result from a clinical trial is sometimes unreliable for guiding clinical practice

When considering whether a patient is likely to benefit from a therapy, the most relevant measure of treatment effect is the absolute risk reduction (ARR) (see Appendix 1) of a treatment (or its reciprocal, the number needed to treat [NNT], [see Appendix 1]) [10,11]. It is well known that a study's overall ARR or NNT will often not reflect a treatment's true ARR for many people in the trial, since a 25% relative risk reduction (RRR) (see Appendix 1) in high risk patients produces much more benefit than it does in low-risk patients (resulting in substantial HTE). For example, Table 1 shows results for a hypothetical treatment that reduces all study subjects' risk by 25%. This results in the overall NNT of 50 greatly underestimating the benefits for high-risk subjects (NNT = 20) and greatly over-estimating the benefits for the typical patient (NNT = 100).

**Table 1:** How summary results of clinical trials can be misleading even when everyone gets the same relative risk reduction.

*Assumption: Treatment reduces baseline risk by 25% without any treatment related harm*					
	Control Event Rate* (CER, %)	**Experimental Event Rate** **(EER, %)**	**Relative Risk Reduction (RRR)**	**Absolute Risk Reduction** **(ARR)**	**Number Needed to Treat (NNT)**
	
(% of study population)					
**Overall result **(100%)	**8**	**6**	**0.25**	**0.02**	**50**
Average risk subjects (75%)	4	3	0.25	0.01	100
High risk subjects (25%)	20	15	0.25	0.05	20

* See Appendix 1

Indeed, because a minority of high-risk patients may account for most trial adverse outcomes and because even a small degree of treatment-related harm can nullify or outweigh benefits in low risk patients, it does not take extreme assumptions to produce scenarios in which almost all individuals [6,12,13] in the trial have an ARR that is substantially lower than that suggested by the summary results reported in the trial. For example, Table 2 shows results that would emerge if the treatment reduces disease-related risk by 25% (just like in Table 1) but now also carries a 2 in 1000 risk of a serious treatment-related harm (due to adverse events or major side-effects). In Scenario #1, the clinical trial's overall result suggests that the treatment has a moderate benefit (RRR = 12.5% and NNT = 100), despite the fact that 75% of study subjects received absolutely no net benefit (i.e. treatment-related harm equals treatment benefit). In Scenario #2, we see that if the difference between outcome risks of low vs. high risk patients is increased (i.e. risk strata more dissimilar in risk), the summary results can still suggest an overall benefit of treatment even though the treatment risks out-weigh treatment benefits for 75% of study subjects (Table 2).

**Table 2:** How summary results can obscure situations where the typical patient receives no benefit or harm

*Assumption: Treatment reduces baseline risk by 25% but with a cost of 2 serious treatment-related adverse events per 1,000 patients per year*					
	**Control Event Rate** **(CER, %)**	**Experimental Event Rate** **(EER, %)**	**Relative Risk Reduction (RRR)**	**Absolute Risk Reduction** **(ARR)**	**Number Needed to Treat (NNT)**
	
(% of study population)	Results over 5 years				
**Scenario #1**					
**Overall result **(100%)	**8**	**7**	**0.125**	**0.01**	**100**
Average risk subjects (75%)	4	4	0	0	∞
High risk subjects (25%)	20	16	0.2	0.04	20
					
**Scenario #2**					
**Overall result **(100%)	**9**	**7.75**	**0.14**	**0.0125**	**80**
Average risk subjects (75%)	2	2.5	-0.25†	-0.005†	-200
High risk subjects (25%)	30	23.5	0.22	0.065	15

† The minus sign denotes that treatment had net harm, rather than benefit.

While these examples illustrate cases in which the absence of risk-based analysis will result in harmful (or merely wasteful) over-treatment, under certain circumstances the opposite may also be the case; a treatment's effect may be null overall, even though it provides substantial benefit in a patient subgroup (typically at high risk for the outcome of interest or at especially low risk of treatment-related harm) [14,15].

### Why risk stratified analyses should be performed whenever feasible

Although the degree of heterogeneity in risk shown in Tables 1 and 2 may seem extreme, such variability in risk is actually quite common when risk-heterogeneity is assessed using a multivariable prediction tool. It has been documented that outcome rates in the highest risk quartile (the 25% of study subjects with the highest predicted risk) in large clinical trials is often 5-20 times higher than in the lowest risk quartile [5,16-20]. While the degree of risk heterogeneity may vary across medical domains, multiple independent risk factors exist for virtually any clinical outcome that would be the target of a therapeutic trial, and therefore, substantial risk heterogeneity should be common. In turn, the presence of risk heterogeneity mathematically implies the presence of HTE, on the absolute risk scale, regardless of whether there is also HTE on the relative risk scale.

Recent research has demonstrated that, even when there are large and clinically important differences in treatment effects across risk groups, conventional subgroup analyses (which assess HTE "one-variable-at-a-time") are inadequate to detect these differences across risk subgroups because they do not account for the fact that patients have multiple variables that determine risk simultaneously [6,9,21-24]. Instead, they examine treatment effect differences based on groups differing on only a single variable, falsely determining a "consistency of treatment effect" across subgroups simply because the groups compared are more similar than dissimilar. Additionally, because conventional subgroup analyses involve multiple comparisons and involve splitting the overall sample to smaller sub-samples, they are both under-powered for detecting genuine subgroup effects (prone to false-negatives), and even more commonly they are prone to false positive findings [25-31]. Clinical trials, so analyzed, can thus result in treatment recommendations and guidelines that promote substantial over- and under-treatment.

There are better alternatives to one-variable-at-a-time subgroup analyses. Multivariable subgroup analysis is theoretically possible, and has been shown to be potentially useful[5], but statistical power is usually inadequate in anything other than pooled analyses of data from multiple trials. Risk-based analyses using multivariable risk prediction tools are more often feasible and have a lower risk of false positive findings than single variable subgroup analysis, when employed as a single pre-specified analysis that avoids the multiplicity of comparisons inherent in testing each sub-grouping variable separately[9]. Moreover, such an analysis will often have more optimal statistical power, as it compares patients that differ in multiple important characteristics simultaneously. Otherwise undetected yet clinically meaningful differences in relative treatment benefit have been demonstrated in many areas where multivariate risk-based approaches have been applied, most particularly in the areas of cardiovascular and cerebrovascular disease, but others as well (Table 3).

**Table 3:** Examples of Clinically Important Risk-based Heterogeneity of Treatment Effect

Clinical Condition	Treatments	Findings
Symptomatic carotid stenosis	Carotid endarterectomy (CEA)	While overall results showed CEA to reduce stroke risk in patients with severe stenosis, risk-benefit stratification demonstrated that benefit is limited to those with high risk features, but without risks factors for perioperative complications[21].

		

Non-valvular atrial fibrillation (AF)	Anticoagulation for primary prevention of stroke	While warfarin prevents stroke in patients with AF compared to aspirin, patients without risk factors for stroke do not benefit incrementally[49,50].

		

Coronary artery disease (CAD)	Coronary artery bypass grafting (CABG)	Early coronary artery bypass grafting reduces total mortality compared to medical therapy in medium and high risk patients, while low risk patients have a non-significant trend toward increased mortality[64].

		

Primary prevention of coronary artery disease	Lipid lowering	Statin therapy reduced risk of myocardial infarction or death, but low risk patients are highly unlikely to benefit despite hyperlipidemia[65].

		

Acute coronary syndromes (ACS)	Early invasive (versus conservative) strategy Enoxaparin (versus unfractionated heparin) Tirofiban (versus placebo)	These therapies reduce the risk of myocardial infarction or death in high risk but not in low risk patients[46-48,66,67]. The risks of bleeding with intensive antithrombotic regimens outweigh benefits in low risk patient. Risk stratification has become central to the management of ACS[68].

		

ST-Elevation acute myocardial infarction	tPA (versus streptokinase) Percutaneous coronary intervention [PCI] (versus thrombolytic therapy)	tPA improves mortality in high risk patients compared to streptokinase, but not in low risk patients. When low risk patients have an excess of risk factors for bleeding, risks of therapy may outweigh benefits[17,55].
		Mortality benefits of PCI are limited to only a relatively limited high risk subgroup [69,70].

		

Severe sepsis	Drotrecogin alfa (activated protein C)	While the pivotal phase III trial demonstrated a significant mortality reduction overall, this was found to be limited only to the half of patients with a high baseline mortality risk. Lower risk patients were exposed to bleeding risks, without a mortality benefit [68,71,72].

### A proposal for reporting clinical trials to provide more information on clinically important heterogeneity in treatment effects (HTE)

Several recent papers have addressed important considerations when conducting and interpreting subgroup analyses [5-7,9,14,22,27,30,32-40], but did not recommend a specific framework for reporting HTE and did not discuss how to deal with multivariable risk analyses. Only a few previous papers have addressed multivariable risk analyses. Herein, we propose some practical guidance for when and how such analyses should be performed and presented (summarized in Appendix 2). While this framework has not been subjected to a formal consensus building process involving a broad sample of stakeholders and is therefore provisional, the approach is a synthesis of ideas and contributions made by many investigators [4-7,9,14,16,17,27,41], and is proposed to provide a considered basis for subsequent discussion, revision, and refinement.

### Recommendation #1: Evaluate and report on the distribution of baseline risk in the overall study population and in the separate treatment arms of the study by using a risk prediction tool

Although its importance was highlighted over a decade ago[12], reporting the distribution of baseline risk (see Appendix 1) is rarely done. Therefore, it is generally impossible to assess the degree of baseline risk heterogeneity in most published clinical trials, since risk heterogeneity cannot be determined when each risk factor's prevalence is listed individually.

The precise approach for presentation is not important, as long as it allows the reader to understand the distribution of predicted baseline risk (or the risk score of a risk index) in the study population. "Table 1" of a clinical trial report (which conventionally includes patient attributes for those in the different study arms) should include, at minimum, the population mean (+ SD) and median predicted baseline risk (or risk score), and additional information on the population distribution if there is substantial skew in subject risk (such as quartiles/percentiles, a histogram or a box plot) (see Table 4). If the study includes a largely homogeneous population with regard to overall risk, the reader will know that generalizing the study results to those with substantially different risk would be speculative. If there is substantial heterogeneity in the study population, then reviewers will know that risk stratified analysis is particularly important.

**Table 4:** Presenting the distribution of baseline risk in clinical trials

	Frequency (%)		
Predicted Risk*	**Control** **(N = 200)**	**Intervention** **(N = 200)**	**Total** **(N = 400)**

< 5%	69 (34.5%)	69 (34.5%)	138 (34.5%)

5%-15%	90 (45.0%)	95 (47.5%)	185 (46.3%)

> 15%	41 (20.5%)	36 (18.0%)	77 (19.3%)

			

Mean + SD	9.2 (8.6)	9.8 (9.3)	9.5 (9.0)

Median (Q_1 _- Q_3_)	6.4 (3.7-10.9)	7.0 (3.6-11.9)	6.8 (3.6-11.3)

EQuRR**	-	-	12.4

* Presenting results so that reader can easily observe whether the relative risk reduction or number needed to treat vary based upon the individuals baseline risk of the outcome. In this example, the risk model is expressed as predicted risk (%). However, presentation of results stratified according to a risk score would be similarly informative.

** Extreme quartile risk ratio, the predicted risk in the highest risk quartile divided by the risk in the lower risk quartile

Finally, including this information in "Table 1" of a clinical trial allows the reader to assess whether there are important baseline differences between treatment arms on the most important baseline attribute (i.e., differences in overall risk for the study's main outcome). It is common to note multiple modest deviations between treatment arms when baseline patient factors are listed one at a time. These differences typically have little influence on trial results, particularly when they combine so as to cancel each other out. However, similar differences in overall baseline risk may influence the trial result, such that comparing the risk distribution between the treatment groups using a composite risk model can be informative and facilitate risk adjustment.

### Recommendation #2: Report how relative and absolute risk reduction varies by baseline risk, using a multivariable prediction tool

There are two fundamental reasons why all clinical trials should attempt to assess how net treatment benefit and safety vary as a function of predicted untreated risk: 1) It allows us to understand how *absolute *risk reduction varies across the study population even when *relative *risk reduction is constant (see Table 1); and 2) net relative risk reduction may not be constant across risk groups, particularly if there is even a small amount of treatment-related harm (see Table 2). For major clinical trials (those that assess a treatment's effect on mortality and major morbidity), it is usually possible to perform risk-based analysis of HTE using an externally developed tool, since prediction tools to estimate overall risk have been developed for most major conditions and their complications (including cardiac, cancer, stroke, renal failure, ICU and hospital morality, etc [see Additional file 1]). Testing risk-based HTE using internally-developed models (based on a blinded regression analysis of the data using all treatment arms) may be useful when such models do not exist. However, when available, we favor the use of an *externally *developed prediction model since over-fitting can potentially exaggerate the degree of risk heterogeneity.

In reporting risk stratified results, readers should be provided with the information needed to easily determine the amount of variation in ARR/NNT and RRR. An approach to presenting these results to a general readership is shown in Table 5. How statistical testing for HTE should be addressed, including for multivariable risk-stratified analyses, is discussed below (Recommendation #5).

**Table 5:** Presenting results showing heterogeneity in treatment effect (HTE)*

	Weighted Event Rate		Relative Risk Reduction	Number Needed to Treat
Predicted Risk*	Control	Intervention	(95% CI)	
< 5%	15/428 (3.5%)	17/431 (3.9%)	-13% (-122%, 43%))**	-250**
				
5%-15%	66/581 (11.4%)	48/580 (8.3%)	27% (-4%, 49%)	32
> 15%	66/310 (21.3%)	38/307 (12.4%)	42% (16%, 60%)	11
				
Overall	147/1319 (11.1%)	103/1318 (7.8%)	30% (11%, 45%)	30

* Although the predicted baseline risk can be shown in categories, the statistical testing of HTE should usually be based upon the full continuous variable. If standard predicted risk categories have been previously proposed in the validated prediction model, this should be stated, referenced appropriately, and clarified why these risk categories make sense (e.g. thresholds for deciding on whether some standard treatment is indicated, uncertain, or not indicated).

** Negative sign denotes net harm, denoting relative risk increase or number need to harm.

### Recommendation #3: Additional primary subgroup analysis for single variables should be pre-specified and limited to patient attributes with strong a priori pathophysiological or empirical justification

Here we define *primary subgroup analysis *as those subgroup comparisons that are well justified (hypothesis-testing, not hypothesis-generating) so as to yield potentially actionable results appropriate for guiding clinical care. Therefore, all primary subgroup comparisons must be fully specified and justified *a priori*.

The number of comparisons made in the primary subgroup analysis should be kept small in number to minimize false positive results, since each additional subgroup comparison decreases the usefulness of the other primary subgroup analyses and should therefore exact a statistical penalty (see recommendation #5). Often, no single variable subgroup analysis (such as by age, by sex, by race, etc.) will be indicated as part of the primary subgroup analysis. Rather, these should generally be conducted as exploratory (secondary) analyses (see recommendation #4), unless: 1) there exists previous empirical evidence from observational studies or exploratory subgroup analyses in prior clinical trials; or 2) there are highly compelling reasons to believe the patient attribute is likely to importantly influence the relative treatment effect (such as time to treatment with time-sensitive therapies or biomarkers that are strong candidates to be specific targets of therapy [e.g. estrogen receptor positivity in breast cancer]).

Prespecification of primary subgroups should include explicit definitions and categories of the subgroup variables, including cut-off thresholds for continuous or ordinal variables where these are used, and the anticipated direction of the effect modification. While it is ideal that analyses should be pre-specified at the time of trial initiation [22,27], it is most important that all primary subgroup analyses be pre-specified prior to examination of the data to ensure that analyses are not biased by multiple comparisons, including *post-hoc *changes in variable construction to better "fit the data". By conducting primary subgroup analysis that are few in number, fully pre-specified, hypothesis-driven and more statistically robust (see recommendation #5), examinations of HTE can produce strong and actionable evidence regarding which patients are most likely to benefit from treatment.

### Recommendation #4: Secondary (exploratory) subgroup analyses should be clearly distinguished from primary subgroup comparisons

Although we propose making a clear distinction between primary and secondary subgroup analyses, it would be a mistake to forgo secondary analyses. Secondary analyses can explore evidence of unexpected relationships between individual patient attributes and treatment effects. Although exploratory analyses are an important part of scientific discovery, it is critically important to understand that such analyses are mainly appropriate for hypotheses generation, which can then be tested (and usually disproved) in future studies. Although medical journals may be reluctant to report "exploratory" analyses, it would be quite easy to routinely include secondary subgroup analyses in an electronic appendix to be published online with the main results of a clinical trial, making them available to the scientific community and for future meta-analyses while keeping them distinct from the primary results.

### Recommendation # 5 All analyses conducted must be reported and statistical testing of HTE should be done using appropriate methods (such as interaction terms) and avoiding overinterpretation

Reporting must include results for *all *subgroup analyses, including multivariate-risk, primary and secondary subgroup analyses, and the paper must state that the primary subgroup analyses conducted were pre-specified. Because statistically significant benefit is likely to be absent in small subgroups, the correct analysis is not to test the significance of the treatment effect in one subgroup or another, but whether the effect differed significantly between subgroups. Work by Brookes et al suggests that the most statistically robust approach to assessing HTE is using interaction terms in regression models [22,23]. Further, they found that testing continuous variables (such as baseline LDL level) is substantially more statistically powerful than testing categorical variables (such as baseline LDL < 100 vs. 100-145 vs > 145). Therefore, unless there is reason to believe that an effect is non-linear, HTE of continuous effects should be tested using the full power of the continuous variable, although categorical results can be shown for simplified presentation in the results section (see Table 5).

Where formal statistical testing fails to detect heterogeneity on the relative risk scale, the conservative assumption of a constant relative risk reduction across all risk groups may generally apply, especially if the study is large enough so that the test for interaction is adequately-powered. One should beware of the remaining possibility of false-negatives (as well as false-positives), especially in underpowered settings. Therefore interpretation of interaction effects should be cautious and viewed also in the context of additional prior/external evidence.

Results of subgroup analyses should be presented so that ARR/NNT as well as RRR can be assessed across risk categories or other subgroups. For instances where multiple single-variable subgroup analyses are performed as part of the primary subgroup analysis, the significance threshold should be adjusted for multiple testing[42,43]..

### Caveats and Future Work

Ideally, a continually updated registry containing easily-applicable, well-accepted, well-validated prediction tools for all the primary clinical outcomes used in trials for all major medical conditions would be available. We recognize that this is not currently the case and that the state of the predictive modeling literature is far from this ideal even for fields that have a long tradition in predictive modeling[44,45]. However, while there is not a well-accepted and validated prediction tool appropriate for every condition, it is important to understand that testing for evidence for HTE using a risk-stratified analysis is a much easier task than determining how risk-stratification should be used in clinical practice. Recent research has demonstrated that a risk prediction tool of even moderate predictive power can typically provide adequate statistical power for answering the scientific question of whether there is evidence that the RRR of treatment varies significantly as a function of baseline risk [9]. It has been shown that even a relatively mediocre prediction tool (AUROC .6 to .65) can substantially improve statistical power over that achieved by examining even strong single risk factors one at a time to test for the presence of risk-based HTE [9]. Indeed, several commonly used scores, such as the Thrombolysis in Myocardial Infarction (TIMI) risk score (for acute coronary syndrome) and CHADS_2 _score (for non-valvular atrial fibrillation), have discriminatory power in this range but have nevertheless proved useful in the detection of risk-based HTE (see Table 3) [46-50].

Moreover, for many fields, it is likely that the widely-accepted predictive models will not be stable but will continuously improve with the addition of new informative predictors (e.g. previously unrecognized genetic risk factors). One may conceive the possibility of re-analyses of the results of clinical trials using more informative prediction models if and when such additional information has been collected. Such re-analyses need to follow equally robust standards as we noted above for the original risk stratification analyses.

For trials that do not have adequate outcome prediction tools to use, risk tools can often be developed on pre-existing data in the trial planning phase, or prior to analysis. Use of internally developed risk models has been advocated [16,51,52] and several large trials have used this approach as the basis for testing risk-based HTE [53-55]. Future work should explore the degree to which over-fitting may bias such an approach and, if so, how best to avoid this. Regardless of the approach, in most instances in which a risk-based analysis shows significant HTE, the finding will be a call for rigorous follow-up research to assess and optimize clinically-feasible risk prediction.

Other medical conditions may have multiple models that might yield clinically different results, frequently on the individual patient-level (where clinical recommendations may be altered depending on which model is used) and sometimes regarding the presence or absence of HTE overall. While future work is needed to address this issue, it should be noted that the ambiguity about how best to treat individuals in such cases is revealed, not created, by risk-based analysis.

This paper has focused exclusively on binary outcomes. Continuous outcomes can be approached with similar principles regarding testing for HTE, as well as primary and secondary subgroup analyses, but obviously metrics such as ARR and RRR would need to be replaced by absolute and relative changes in the continuous measure of interest; and NNT is not pertinent to continuous outcomes, unless the continuous measures are grouped into justifiable binary categories.

Additionally, we focused on heterogeneity in the dimension of outcome risk; other risk dimensions may also be important, such as the risk of treatment-related harm (for therapies with serious and common adverse events) [15] or competing risk (especially for conditions including many patients with multiple morbidities or older patients in trials measuring longer-term outcomes) [8,56-58]. Multivariate models predicting treatment-related adverse events, such as those developed to predict anticoagulant- or thrombolytic-related serious bleeding [59,60] or surgical risks for specific procedures, may be useful in the first case, and comorbidity indices [56,61] in the second. There are also examples where combining models for treatment-related harm with outcome risk models to stratify trial results using a risk-benefit scheme has yielded informative results [17,21]. However, whether, when, and how to perform these complex analyses are methodologically fraught issues that may be difficult to make routine recommendations on.

As we and others have noted elsewhere, we will never be able to get all the information needed for informing clinical practice and health policy from experimental trials [5,27-29,62,63]. The approach we outline here may not be applicable or feasible for many trials, particularly early phase trials, which tend to be small and explanatory in nature, and often use surrogate instead of clinical endpoints. Furthermore, the above suggestions only deal with assessing HTE statistically in the context of trials and not how best to promote the use of risk stratification in clinical practice. Despite these caveats and limitations, for pivotal, phase III clinical trials using clinically important outcomes, the suggested approach should usually be feasible and should substantially improve our ability to produce scientifically valid information on HTE to better inform clinical practice.

## Conclusion

*Implications for the peer-review and publishing of clinical trials*

While it is well appreciated that outcome risk heterogeneity is common and can lead to clinically meaningful HTE, few clinical trials analyze the variation in treatment effect across the spectrum of patients in their studies and subgroup analyses are performed and reported erratically [14,30,33,35]. Though some argue that journals should not dictate the scientific questions that investigators address, for many important trials, the results are not fully disclosed in the absence of a risk-based analysis. While risk-stratified results may emphasize the importance of treatment in high-risk patients and may even result in the discovery of patient sub-groups who benefit when summary results of trials are negative, such analyses may be particularly resisted when trial results are overall positive, given the obvious incentives for industry to get treatments approved for as broad a population as possible [14]. There also exist incentives to selectively highlight positive exploratory subgroup analyses, when overall results are negative. Therefore, it seems likely that inadequate investigation and reporting of HTE will continue to be a problem unless editors, granting agencies and government regulators insist upon it. Suggestions herein provide a framework for the development of implementable guidelines that might support routine examination and reporting of information essential for optimizing medical care for individuals.

## Competing interests

Dr Kent has received research funding from Pfizer, Inc.

## Authors' contributions

All authors contributed to the conceptual framework presented in the manuscript. DMK and RAH co-wrote the initial draft. All authors revised the manuscript for important content and approved the final manuscript.

## Appendix

### Appendix 1. Glossary

#### Baseline Risk

Risk of a particular event (in this paper, typically the primary study outcome) in the absence of the experimental therapy.

#### Event rate

Proportion or percentage of study participants in a group in which a particular event (typically the primary outcome) is observed. **Control event rate **(CER) and **experimental event rate **(EER) are used to refer to event rates in the control group and experimental group, respectively. In a clinical trial, baseline risk is best estimated by the observed control event rate (CER).

#### Relative Risk Reduction (RRR)

The proportional reduction in the rate of bad events between experiment (experimental event rate [EER]) and control (control event rate [CER]) patients in a trial, calculated as (CER - EER)/CER. Moreover, we use the term "net RRR" in this paper to emphasize that we are assessing the overall treatment benefit (treatment-related benefit *minus *treatment-related harm). This is merely the RRR when outcome measure is a composite of all major outcomes related to the treatment, both those that are decreased and those that are increased by treatment. For parsimony, we consider here that all outcomes have similar importance, but this may not necessarily by generalizable (e.g. many composite outcomes in the literature are a conglomerate of endpoints with very different connotations and clinical importance).

#### Absolute Risk Reduction (ARR)

The absolute arithmetic difference in event rates between the control group and the experimental group (CER - EER).

#### Number Needed to Treat (NNT)

The number of patients who need to be treated, on average, to prevent 1 additional bad outcome; calculated as 1/ARR.

### Appendix 2. Checklist for Reporting on Subgroup Analyses & Heterogeneity in Treatment Effects

*1. Evaluate and report on the distribution of risk in the overall study population and in the separate treatment arms of the study by using a risk prediction model or index*.

• Report on the distribution of predicted risk (or risk score) in the study population overall and by treatment arm.

• Risk reporting should allow readers to assess the full distribution of the study population either graphically (e.g., histograms or box & whiskers plots) or by including information on the mean, standard deviation, median and interquantile ranges.

*2. Primary subgroup analyses should include reporting how relative and absolute risk reduction varies in a risk-stratified analysis*.

• The risk prediction model should be pre-specified (i.e., fully specified before *any *analysis of treatment-effect has begun) and preferably externally developed.

• Both absolute and relative risk reductions must be reported.

*3. Any additional primary subgroup analysis should be pre-specified and limited to patient attributes with strong *a priori *pathophysiological or empirical justification*.

• All primary subgroup comparisons must be pre-specified.

• Prespecification should include all aspects of the subgroup analysis, including threshold values for continuous or ordinal variables where these are used.

• All primary subgroup analyses must be justified based upon pathophysiological or empirical evidence that this factor modifies treatment effects.

*4. Conduct and report on secondary (exploratory) subgroup analyses separately from primary subgroup comparisons*.

• Secondary subgroup analyses must be reported separately from primary subgroup analyses and clearly labeled as exploratory (potential useful for hypothesis generation and informing future research, but having little or no immediate relevance to patient care).

*5. All analyses conducted must be reported and statistical testing of HTE should be done using appropriate methods (such as interaction terms) and avoiding overinterpretation*.

• Reporting must include results for all subgroup analyses conducted and the paper must state that primary subgroup analyses conducted were pre-specified and reported.

• Statistical comparisons should be limited to reporting for statistical significance of treatment heterogeneity between subgroups using interaction terms. (Testing for the significance of a treatment effect within a subgroup is inappropriate due to poor statistical power).

• Statistical comparisons should be corrected for the number of primary subgroup analyses performed.

## Supplementary Material

Additional file 1 **Predictive models for some commonly used outcomes in clinical trials; references for 95 prognostic models**. Click here for file

## References

[B1] Black D The limitations of evidence J R Coll Physicians Lond 1998 32 23 26 9507437 PMC9662961

[B2] Feinstein AR Horwitz RI Problems in the "evidence" of "evidence-based medicine" Am J Med 1997 103 529 535 10.1016/S0002-9343(97)00244-1 9428837

[B3] Caplan LR Evidence based medicine: concerns of a clinical neurologist J Neurol Neurosurg Psychiatry 2001 71 569 574 10.1136/jnnp.71.5.569 11606661 PMC1737598

[B4] Rothwell PM Can overall results of clinical trials be applied to all patients? Lancet 1995 345 1616 1619 10.1016/S0140-6736(95)90120-5 7783541

[B5] Rothwell PM Mehta Z Howard SC Gutnikov SA Warlow CP Treating individuals 3: from subgroups to individuals: general principles and the example of carotid endarterectomy Lancet 2005 365 256 265 15652609 10.1016/S0140-6736(05)17746-0

[B6] Kent DM Hayward RA Limitations of applying summary results of clinical trials to individual patients: the need for risk stratification JAMA 2007 298 1209 1212 10.1001/jama.298.10.1209 17848656

[B7] Kravitz RL Duan N Braslow J Evidence-based medicine, heterogeneity of treatment effects, and the trouble with averages Milbank Q 2004 82 661 687 10.1111/j.0887-378X.2004.00327.x 15595946 PMC2690188

[B8] Kent DM Alsheikh-Ali AA Hayward RA Competing risk and heterogeneity of treatment effect in clinical trials Trials 2008 9 30 10.1186/1745-6215-9-30 18498644 PMC2423182

[B9] Hayward RA Kent DM Vijan S Hofer TP Multivariable risk prediction can greatly enhance the statistical power of clinical trial subgroup analysis BMC Med Res Methodol 2006 6 18 10.1186/1471-2288-6-18 16613605 PMC1523355

[B10] Ebrahim S Smith GD The 'number need to treat': does it help clinical decision making? J Hum Hypertens 1999 13 721 724 10.1038/sj.jhh.1000919 10578213

[B11] Furukawa TA Guyatt GH Griffith LE Can we individualize the 'number needed to treat'? An empirical study of summary effect measures in meta-analyses Int J Epidemiol 2002 31 72 76 10.1093/ije/31.1.72 11914297

[B12] Ioannidis JP Lau J The impact of high-risk patients on the results of clinical trials J Clin Epidemiol 1997 50 1089 1098 10.1016/S0895-4356(97)00149-2 9368516

[B13] Glasziou PP Irwig LM An evidence based approach to individualising treatment BMJ 1995 311 1356 1359 7496291 10.1136/bmj.311.7016.1356 PMC2551234

[B14] Hayward RA Kent DM Vijan S Hofer TP Reporting clinical trial results to inform providers, payers, and consumers Health Aff (Millwood) 2005 24 1571 1581 10.1377/hlthaff.24.6.1571 16284031

[B15] Kent DM Ruthazer R Selker HP Are some patients likely to benefit from recombinant tissue-type plasminogen activator for acute ischemic stroke even beyond 3 hours from symptom onset? Stroke 2003 34 464 467 10.1161/01.STR.0000051506.43212.8B 12574561

[B16] Ioannidis JP Lau J Heterogeneity of the baseline risk within patient populations of clinical trials: a proposed evaluation algorithm Am J Epidemiol 1998 148 1117 1126 9850135 10.1093/oxfordjournals.aje.a009590

[B17] Kent DM Hayward RA Griffith JL Vijan S Beshansky JR Califf RM Selker HP An independently derived and validated predictive model for selecting patients with myocardial infarction who are likely to benefit from tissue plasminogen activator compared with streptokinase Am J Med 2002 113 104 111 10.1016/S0002-9343(02)01160-9 12133748

[B18] Kent DM Ruthazer R Griffith JL Beshansky JR Grines CL Aversano T Concannon TW Zalenski RJ Selker HP Comparison of mortality benefit of immediate thrombolytic therapy versus delayed primary angioplasty for acute myocardial infarction Am J Cardiol 2007 99 1384 1388 10.1016/j.amjcard.2006.12.068 17493465

[B19] Kent DM Jafar TH Hayward RA Tighiouart H Landa M de Jong P de Zeeuw D Remuzzi G Kamper AL Levey AS Progression risk, urinary protein excretion, and treatment effects of angiotensin-converting enzyme inhibitors in nondiabetic kidney disease J Am Soc Nephrol 2007 18 1959 1965 10.1681/ASN.2006101081 17475813

[B20] Trikalinos TA Ioannidis JP Predictive modeling and heterogeneity of baseline risk in meta-analysis of individual patient data J Clin Epidemiol 2001 54 245 252 10.1016/S0895-4356(00)00311-5 11223322

[B21] Rothwell PM Warlow CP Prediction of benefit from carotid endarterectomy in individual patients: a risk-modelling study. European Carotid Surgery Trialists' Collaborative Group Lancet 1999 353 2105 2110 10.1016/S0140-6736(98)11415-0 10382694

[B22] Brookes ST Whitley E Peters TJ Mulheran PA Egger M Davey SG Subgroup analyses in randomised controlled trials: quantifying the risks of false-positives and false-negatives Health Technol Assess 2001 5 1 56 11701102 10.3310/hta5330

[B23] Brookes ST Whitely E Egger M Smith GD Mulheran PA Peters TJ Subgroup analyses in randomized trials: risks of subgroup-specific analyses; power and sample size for the interaction test J Clin Epidemiol 2004 57 229 236 10.1016/j.jclinepi.2003.08.009 15066682

[B24] Albert JM Gadbury GL Mascha EJ Assessing treatment effect heterogeneity in clinical trials with blocked binary outcomes Biom J 2005 47 662 673 10.1002/bimj.200510157 16385907

[B25] Furberg CD Byington RP What do subgroup analyses reveal about differential response to beta-blocker therapy? The Beta-Blocker Heart Attack Trial experience Circulation 1983 67 I98 101 6133654

[B26] Tannock IF False-positive results in clinical trials: multiple significance tests and the problem of unreported comparisons J Natl Cancer Inst 1996 88 206 207 10.1093/jnci/88.3-4.206 8632495

[B27] Rothwell PM Treating individuals 2. Subgroup analysis in randomised controlled trials: importance, indications, and interpretation Lancet 2005 365 176 186 10.1016/S0140-6736(05)17709-5 15639301

[B28] Assmann SF Pocock SJ Enos LE Kasten LE Subgroup analysis and other (mis)uses of baseline data in clinical trials Lancet 2000 355 1064 1069 10.1016/S0140-6736(00)02039-0 10744093

[B29] Oxman AD Guyatt GH A consumer's guide to subgroup analyses Ann Intern Med 1992 116 78 84 1530753 10.7326/0003-4819-116-1-78

[B30] Hernandez AV Boersma E Murray GD Habbema JD Steyerberg EW Subgroup analyses in therapeutic cardiovascular clinical trials: are most of them misleading? Am Heart J 2006 151 257 264 10.1016/j.ahj.2005.04.020 16442886

[B31] Ioannidis JP Why most published research findings are false PLoS Med 2005 2 e124 10.1371/journal.pmed.0020124 16060722 PMC1182327

[B32] Feiveson AH Power by simulation The Stata Journal 2009 2 107 124

[B33] Wang R Lagakos SW Ware JH Hunter DJ Drazen JM Statistics in medicine--reporting of subgroup analyses in clinical trials N Engl J Med 2007 357 2189 2194 10.1056/NEJMsr077003 18032770

[B34] Yusuf S Wittes J Probstfield J Tyroler HA Analysis and interpretation of treatment effects in subgroups of patients in randomized clinical trials JAMA 1991 266 93 98 10.1001/jama.266.1.93 2046134

[B35] Parker AB Naylor CD Subgroups, treatment effects, and baseline risks: some lessons from major cardiovascular trials Am Heart J 2000 139 952 961 10.1067/mhj.2000.106610 10827374

[B36] Pocock SJ Assmann SE Enos LE Kasten LE Subgroup analysis, covariate adjustment and baseline comparisons in clinical trial reporting: current practice and problems Stat Med 2002 21 2917 2930 10.1002/sim.1296 12325108

[B37] Kraemer HC Frank E Kupfer DJ Moderators of treatment outcomes: clinical, research, and policy importance JAMA 2006 296 1286 1289 10.1001/jama.296.10.1286 16968853

[B38] Davidoff F Heterogeneity is not always noise: lessons from improvement JAMA 2009 302 2580 2586 10.1001/jama.2009.1845 20009058

[B39] Gabler NB Duan N Liao D Elmore JG Ganiats TG Kravitz RL Dealing with heterogeneity of treatment effects: is the literature up to the challenge? Trials 2009 10 43 10.1186/1745-6215-10-43 19545379 PMC2706823

[B40] Sun X Briel M Walter SD Guyatt GH Is a subgroup effect believable? Updating criteria to evaluate the credibility of subgroup analyses BMJ 2010 340 c117 10.1136/bmj.c117 20354011

[B41] Greenfield S Kravitz R Duan N Kaplan SH Heterogeneity of treatment effects: implications for guidelines, payment, and quality assessment Am J Med 2007 120 S3 S9 10.1016/j.amjmed.2007.02.002 17403380

[B42] Proschan MA Waclawiw MA Practical guidelines for multiplicity adjustment in clinical trials Control Clin Trials 2000 21 527 539 10.1016/S0197-2456(00)00106-9 11146147

[B43] Bender R Lange S Adjusting for multiple testing--when and how? J Clin Epidemiol 2001 54 343 349 10.1016/S0895-4356(00)00314-0 11297884

[B44] Tzoulaki I Liberopoulos G Ioannidis JP Assessment of claims of improved prediction beyond the Framingham risk score JAMA 2009 302 2345 2352 10.1001/jama.2009.1757 19952321

[B45] Ioannidis JP Tzoulaki I What makes a good predictor?: the evidence applied to coronary artery calcium score JAMA 2010 303 1646 1647 10.1001/jama.2010.503 20424257

[B46] Antman EM Cohen M Bernink PJ McCabe CH Horacek T Papuchis G Mautner B Corbalan R Radley D Braunwald E The TIMI risk score for unstable angina/non-ST elevation MI: A method for prognostication and therapeutic decision making JAMA 2000 284 835 842 10.1001/jama.284.7.835 10938172

[B47] Morrow DA Antman EM Snapinn SM McCabe CH Theroux P Braunwald E An integrated clinical approach to predicting the benefit of tirofiban in non-ST elevation acute coronary syndromes. Application of the TIMI Risk Score for UA/NSTEMI in PRISM-PLUS Eur Heart J 2002 23 223 229 10.1053/euhj.2001.2738 11792137

[B48] Cannon CP Weintraub WS Demopoulos LA Vicari R Frey MJ Lakkis N Neumann FJ Robertson DH DeLucca PT DiBattiste PM Gibson CM Braunwald E TACTICS (Treat Angina with Aggrastat and Determine Cost of Therapy with an Invasive or Conservative Strategy)--Thrombolysis in Myocardial Infarction 18 Investigators Comparison of early invasive and conservative strategies in patients with unstable coronary syndromes treated with the glycoprotein IIb/IIIa inhibitor tirofiban N Engl J Med 2001 344 1879 1887 10.1056/NEJM200106213442501 11419424

[B49] Gage BF Waterman AD Shannon W Boechler M Rich MW Radford MJ Validation of clinical classification schemes for predicting stroke: results from the National Registry of Atrial Fibrillation JAMA 2001 285 2864 2870 10.1001/jama.285.22.2864 11401607

[B50] Gage BF van Walraven C Pearce L Hart RG Koudstaal PJ Boode BS Petersen P Selecting patients with atrial fibrillation for anticoagulation: stroke risk stratification in patients taking aspirin Circulation 2004 110 2287 2292 10.1161/01.CIR.0000145172.55640.93 15477396

[B51] Pocock SJ Lubsen J More on subgroup analyses in clinical trials N Engl J Med 2008 358 2076 2077 10.1056/NEJMc0800616 18463389

[B52] Follmann DA Proschan MA A multivariate test of interaction for use in clinical trials Biometrics 1999 55 1151 1155 10.1111/j.0006-341X.1999.01151.x 11315061

[B53] Chen ZM Jiang LX Chen YP Xie JX Pan HC Peto R Collins R Liu LS COMMIT (ClOpidogrel and Metoprolol in Myocardial Infarction Trial) collaborative group Addition of clopidogrel to aspirin in 45,852 patients with acute myocardial infarction: randomised placebo-controlled trial Lancet 2005 366 1607 1621 10.1016/S0140-6736(05)67660-X 16271642

[B54] Yusuf S Diener HC Sacco RL Cotton D Ounpuu S Lawton WA Palesch Y Martin RH Albers GW Bath P Bornstein N Chan BP Chen ST Cunha L Dahlöf B De Keyser J Donnan GA Estol C Gorelick P Gu V Hermansson K Hilbrich L Kaste M Lu C Machnig T Pais P Roberts R Skvortsova V Teal P Toni D VanderMaelen C Voigt T Weber M Yoon BW PRoFESS Study Group Telmisartan to prevent recurrent stroke and cardiovascular events N Engl J Med 2008 359 1225 1237 10.1056/NEJMoa0804593 18753639 PMC2714258

[B55] Califf RM Woodlief LH Harrell FE Jr Lee KL White HD Guerci A Barbash GI Simes RJ Weaver WD Simoons ML Topol EJ Selection of thrombolytic therapy for individual patients: development of a clinical model. GUSTO-I Investigators Am Heart J 1997 133 630 639 10.1016/S0002-8703(97)70164-9 9200390

[B56] Litwin MS Greenfield S Elkin EP Lubeck DP Broering JM Kaplan SH Assessment of prognosis with the total illness burden index for prostate cancer: aiding clinicians in treatment choice Cancer 2007 109 1777 1783 10.1002/cncr.22615 17354226

[B57] Braithwaite RS Concato J Chang CC Roberts MS Justice AC A framework for tailoring clinical guidelines to comorbidity at the point of care Arch Intern Med 2007 167 2361 2365 10.1001/archinte.167.21.2361 18039996 PMC3460384

[B58] Greenfield S Billimek J Pellegrini F Franciosi M De Berardis G Nicolucci A Kaplan SH Comorbidity affects the relationship between glycemic control and cardiovascular outcomes in diabetes: a cohort study Ann Intern Med 2009 151 854 60 20008761 10.7326/0003-4819-151-12-200912150-00005

[B59] Gurwitz JH Gore JM Goldberg RJ Barron HV Breen T Rundle AC Sloan MA French W Rogers WJ Risk for intracranial hemorrhage after tissue plasminogen activator treatment for acute myocardial infarction. Participants in the National Registry of Myocardial Infarction 2 Ann Intern Med 1998 129 597 604 9786806 10.7326/0003-4819-129-8-199810150-00002

[B60] Shireman TI Mahnken JD Howard PA Kresowik TF Hou Q Ellerbeck EF Development of a contemporary bleeding risk model for elderly warfarin recipients Chest 2006 130 1390 1396 10.1378/chest.130.5.1390 17099015

[B61] Charlson M Szatrowski TP Peterson J Gold J Validation of a combined comorbidity index J Clin Epidemiol 1994 47 1245 1251 10.1016/0895-4356(94)90129-5 7722560

[B62] Vijan S Kent DM Hayward RA Are randomized controlled trials sufficient evidence to guide clinical practice in type II (non-insulin-dependent) diabetes mellitus? Diabetologia 2000 43 125 130 10.1007/s001250050017 10672454

[B63] Nallamothu BK Hayward RA Bates ER Beyond the randomized clinical trial: the role of effectiveness studies in evaluating cardiovascular therapies Circulation 2008 118 1294 1303 10.1161/CIRCULATIONAHA.107.703579 18794402

[B64] Yusuf S Zucker D Peduzzi P Fisher LD Takaro T Kennedy JW Davis K Killip T Passamani E Norris R Effect of coronary artery bypass graft surgery on survival: overview of 10-year results from randomised trials by the Coronary Artery Bypass Graft Surgery Trialists Collaboration Lancet 1994 344 563 570 10.1016/S0140-6736(94)91963-1 7914958

[B65] West of Scotland Coronary Prevention Study: identification of high-risk groups and comparison with other cardiovascular intervention trials Lancet 1996 348 1339 1342 10.1016/S0140-6736(96)04292-4 8918276

[B66] Mehta SR Granger CB Boden WE Steg PG Bassand JP Faxon DP Afzal R Chrolavicius S Jolly SS Widimsky P Avezum A Rupprecht HJ Zhu J Col J Natarajan MK Horsman C Fox KA Yusuf S TIMACS Investigators Early versus delayed invasive intervention in acute coronary syndromes N Engl J Med 2009 360 2165 2175 10.1056/NEJMoa0807986 19458363

[B67] Mehta SR Cannon CP Fox KA Wallentin L Boden WE Spacek R Widimsky P McCullough PA Hunt D Braunwald E Yusuf S Routine vs selective invasive strategies in patients with acute coronary syndromes: a collaborative meta-analysis of randomized trials JAMA 2005 293 2908 2917 10.1001/jama.293.23.2908 15956636

[B68] Hillis LD Lange RA Optimal management of acute coronary syndromes N Engl J Med 2009 360 2237 2240 10.1056/NEJMe0902632 19458369

[B69] Kent DM Ruthazer R Griffith JL Beshansky JR Concannon TW Aversano T Grines CL Zalenski RJ Selker HP A percutaneous coronary intervention-thrombolytic predictive instrument to assist choosing between immediate thrombolytic therapy versus delayed primary percutaneous coronary intervention for acute myocardial infarction Am J Cardiol 2008 101 790 795 10.1016/j.amjcard.2007.10.050 18328842

[B70] Thune JJ Hoefsten DE Lindholm MG Mortensen LS Andersen HR Nielsen TT Kober L Kelbaek H Danish Multicenter Randomized Study on Fibrinolytic Therapy Versus Acute Coronary Angioplasty in Acute Myocardial Infarction (DANAMI)-2 Investigators Simple risk stratification at admission to identify patients with reduced mortality from primary angioplasty Circulation 2005 112 2017 2021 10.1161/CIRCULATIONAHA.105.558676 16186438

[B71] Xigris: drotrecogin alfa (activated): PV 3420 AMP 2001 Indianapolis, IN, Eli Lilly & co

[B72] Abraham E Laterre PF Garg R Levy H Talwar D Trzaskoma BL François B Guy JS Brückmann M Rea-Neto A Rossaint R Perrotin D Sablotzki A Arkins N Utterback BG Macias WL Administration of Drotrecogin Alfa (Activated) in Early Stage Severe Sepsis (ADDRESS) Study Group Drotrecogin alfa (activated) for adults with severe sepsis and a low risk of death N Engl J Med 2005 353 1332 1341 10.1056/NEJMoa050935 16192478

