# Supplementary material for: Assessing and reporting heterogeneity in treatment effects in clinical trials: a proposal
Source: Trials. 2010 Aug 12;11:85. doi: 10.1186/1745-6215-11-85 (PMC2928211; doi:10.1186/1745-6215-11-85)
Supplement: Additional file 1 — Predictive models for some commonly used outcomes in clinical trials; references for 95 prognostic models. [file 1745-6215-11-85-S1.DOC]

**Additional File 1: Predictive models for some commonly used outcomes in clinical trials.**

Prognostic models exist for many, if not most, of the primary outcomes used in clinical trials. This appendix is a partial list of references for predictive models for some common diseases predicting either clinical or surrogate outcomes commonly used as primary outcomes in phase III clinical trials.  To generate this list, we examined all clinical trials published in 2007 in: Journal of the American Medical Association (JAMA); Lancet; BMJ and New England Journal of Medicine, and then performed a pubmed search for a predictive model that applies to the disease-population that predicts the primary study outcome.

The list includes models for cardiovascular disease (including heart failure^1-3^, acute & chronic CAD^4-12^, as well as CHD risk for primary prevention^13-15^); cerebrovascular disease (including the baseline stroke risk for primary prevention^16, 17^, recurrent stroke for secondary prevention^18, 19^, functional outcome in acute stroke^20-22^; stroke following transient ischemic attack^23-25^, risk of stroke with atrial fibrillation^26, 27^); acute and chronic kidney disease^28-30^, oncology models (including breast^31, 31-36^, cervical^37^, colon^38-44^, lung^45-47^, prostate^48-51^, renal^52-55^, hematologic^56-61^, head and neck^62, 63^, gastric^64, 65^, brain^66, 67^, other^68-73^); common endocrine disorders (including the risk of cardiovascular complications in diabetes^74-77^, changes in glycated hemoglobin in diabetes and the risk of osteoporotic fracture^78, 79^), pulmonary and critical care (including ICU mortality^80-83^, in-hospital mortality^84^, COPD^85, 86, 86, 87^, pneumonia^88-90^, sepsis^91^), and other infectious diseases (including HIV^92^ and hepatitis C^93-95^). While it is beyond the scope of this paper to evaluate each of these models individually, many of the included models are well known and have been validated. Thus, during the planning phase of a clinical trial, it is often possible to identify an independently developed model that would be useful to help analyze and interpret trial results.

Reference List

1. O'Connor CM, Abraham WT, Albert NM et al. Predictors of mortality after discharge in patients hospitalized with heart failure: an analysis from the Organized Program to Initiate Lifesaving Treatment in Hospitalized Patients with Heart Failure (OPTIMIZE-HF). Am Heart J 2008; 156(4):662-673.

2. Abraham WT, Fonarow GC, Albert NM et al. Predictors of in-hospital mortality in patients hospitalized for heart failure: insights from the Organized Program to Initiate Lifesaving Treatment in Hospitalized Patients with Heart Failure (OPTIMIZE-HF). J Am Coll Cardiol 2008; 52(5):347-356.

3. Levy WC, Mozaffarian D, Linker DT et al. The Seattle Heart Failure Model: prediction of survival in heart failure. Circulation 2006; 113(11):1424-1433.

4. Daly CA, De SB, Sendon JL et al. Predicting prognosis in stable angina--results from the Euro heart survey of stable angina: prospective observational study. BMJ 2006; 332(7536):262-267.

5. Clayton TC, Lubsen J, Pocock SJ et al. Risk score for predicting death, myocardial infarction, and stroke in patients with stable angina, based on a large randomised trial cohort of patients. BMJ 2005; 331(7521):869.

6. Madan P, Elayda MA, Lee VV, Wilson JM. Predicting major adverse cardiac events after percutaneous coronary intervention: the Texas Heart Institute risk score. Am Heart J 2008; 155(6):1068-1074.

7. Selker HP, Griffith JL, Beshansky JR et al. Patient-specific predictions of outcomes in myocardial infarction for real-time emergency use: a thrombolytic predictive instrument. Ann Intern Med 1997; 127(7):538-556.

8. Moscucci M, Kline-Rogers E, Share D et al. Simple bedside additive tool for prediction of in-hospital mortality after percutaneous coronary interventions. Circulation 2001; 104(3):263-268.

9. Fox KA, Dabbous OH, Goldberg RJ et al. Prediction of risk of death and myocardial infarction in the six months after presentation with acute coronary syndrome: prospective multinational observational study (GRACE). BMJ 2006; 333(7578):1091.

10. Boersma E, Pieper KS, Steyerberg EW et al. Predictors of outcome in patients with acute coronary syndromes without persistent ST-segment elevation. Results from an international trial of 9461 patients. The PURSUIT Investigators. Circulation 2000; 101(22):2557-2567.

11. Halkin A, Singh M, Nikolsky E et al. Prediction of mortality after primary percutaneous coronary intervention for acute myocardial infarction: the CADILLAC risk score. J Am Coll Cardiol 2005; 45(9):1397-1405.

12. Kattan MW. Judging new markers by their ability to improve predictive accuracy. J Natl Cancer Inst 2003; 95(9):634-635.

13. Lindman AS, Veierod MB, Pedersen JI, Tverdal A, Njolstad I, Selmer R. The ability of the SCORE high-risk model to predict 10-year cardiovascular disease mortality in Norway. Eur J Cardiovasc Prev Rehabil 2007; 14(4):501-507.

14. Pocock SJ, McCormack V, Gueyffier F, Boutitie F, Fagard RH, Boissel JP. A score for predicting risk of death from cardiovascular disease in adults with raised blood pressure, based on individual patient data from randomised controlled trials. BMJ 2001; 323(7304):75-81.

15. Ulmer H, Kollerits B, Kelleher C, Diem G, Concin H. Predictive accuracy of the SCORE risk function for cardiovascular disease in clinical practice: a prospective evaluation of 44 649 Austrian men and women. Eur J Cardiovasc Prev Rehabil 2005; 12(5):433-441.

16. Lumley T, Kronmal RA, Cushman M, Manolio TA, Goldstein S. A stroke prediction score in the elderly: validation and Web-based application. J Clin Epidemiol 2002; 55(2):129-136.

17. Hitman GA, Colhoun H, Newman C et al. Stroke prediction and stroke prevention with atorvastatin in the Collaborative Atorvastatin Diabetes Study (CARDS). Diabet Med 2007; 24(12):1313-1321.

18. Kernan WN, Viscoli CM, Brass LM et al. The stroke prognosis instrument II (SPI-II) : A clinical prediction instrument for patients with transient ischemia and nondisabling ischemic stroke. Stroke 2000; 31(2):456-462.

19. Weimar C, Goertler M, Rother J et al. Systemic risk score evaluation in ischemic stroke patients (SCALA): a prospective cross sectional study in 85 German stroke units. J Neurol 2007; 254(11):1562-1568.

20. Kent DM, Selker HP, Ruthazer R, Bluhmki E, Hacke W. The stroke-thrombolytic predictive instrument: a predictive instrument for intravenous thrombolysis in acute ischemic stroke. Stroke 2006; 37(12):2957-2962.

21. Reid JM, Gubitz GJ, Dai D et al. External validation of a six simple variable model of stroke outcome and verification in hyper-acute stroke. J Neurol Neurosurg Psychiatry 2007; 78(12):1390-1391.

22. Counsell C, Dennis M, McDowall M, Warlow C. Predicting outcome after acute and subacute stroke: development and validation of new prognostic models. Stroke 2002; 33(4):1041-1047.

23. Johnston SC, Rothwell PM, Nguyen-Huynh MN et al. Validation and refinement of scores to predict very early stroke risk after transient ischaemic attack. Lancet 2007; 369(9558):283-292.

24. Rothwell PM, Giles MF, Flossmann E et al. A simple score (ABCD) to identify individuals at high early risk of stroke after transient ischaemic attack. Lancet 2005; 366(9479):29-36.

25. Ay H, Arsava EM, Johnston SC et al. Clinical- and imaging-based prediction of stroke risk after transient ischemic attack: the CIP model. Stroke 2009; 40(1):181-186.

26. Wang TJ, Massaro JM, Levy D et al. A risk score for predicting stroke or death in individuals with new-onset atrial fibrillation in the community: the Framingham Heart Study. JAMA 2003; 290(8):1049-1056.

27. Gage BF, van WC, Pearce L et al. Selecting patients with atrial fibrillation for anticoagulation: stroke risk stratification in patients taking aspirin. Circulation 2004; 110(16):2287-2292.

28. Fiaccadori E, Maggiore U, Lombardi M, Leonardi S, Rotelli C, Borghetti A. Predicting patient outcome from acute renal failure comparing three general severity of illness scoring systems. Kidney Int 2000; 58(1):283-292.

29. Bang H, Vupputuri S, Shoham DA et al. SCreening for Occult REnal Disease (SCORED): a simple prediction model for chronic kidney disease. Arch Intern Med 2007; 167(4):374-381.

30. Kshirsagar AV, Bang H, Bomback AS et al. A simple algorithm to predict incident kidney disease. Arch Intern Med 2008; 168(22):2466-2473.

31. Gail MH, Brinton LA, Byar DP et al. Projecting individualized probabilities of developing breast cancer for white females who are being examined annually. J Natl Cancer Inst 1989; 81(24):1879-1886.

32. Chen J, Pee D, Ayyagari R et al. Projecting absolute invasive breast cancer risk in white women with a model that includes mammographic density. J Natl Cancer Inst 2006; 98(17):1215-1226.

33. Barlow WE, White E, Ballard-Barbash R et al. Prospective breast cancer risk prediction model for women undergoing screening mammography. J Natl Cancer Inst 2006; 98(17):1204-1214.

34. Nieto Y, Nawaz S, Shpall EJ, Bearman SI, Murphy J, Jones RB. Long-term analysis and prospective validation of a prognostic model for patients with high-risk primary breast cancer receiving high-dose chemotherapy. Clin Cancer Res 2004; 10(8):2609-2617.

35. Decarli A, Calza S, Masala G, Specchia C, Palli D, Gail MH. Gail model for prediction of absolute risk of invasive breast cancer: independent evaluation in the Florence-European Prospective Investigation Into Cancer and Nutrition cohort. J Natl Cancer Inst 2006; 98(23):1686-1693.

36. Gail MH. The estimation and use of absolute risk for weighing the risks and benefits of selective estrogen receptor modulators for preventing breast cancer. Ann N Y Acad Sci 2001; 949:286-291.

37. Sherman ME, Lorincz AT, Scott DR et al. Baseline cytology, human papillomavirus testing, and risk for cervical neoplasia: a 10-year cohort analysis. J Natl Cancer Inst 2003; 95(1):46-52.

38. Bonithon-Kopp C, Piard F, Fenger C et al. Colorectal adenoma characteristics as predictors of recurrence. Dis Colon Rectum 2004; 47(3):323-333.

39. Jensen P, Krogsgaard MR, Christiansen J. Prognostic model for patients treated for colorectal adenomas with regard to development of recurrent adenomas and carcinoma. Eur J Surg 1996; 162(3):229-234.

40. Ouellette JR, Small DG, Termuhlen PM. Evaluation of Charlson-Age Comorbidity Index as predictor of morbidity and mortality in patients with colorectal carcinoma. J Gastrointest Surg 2004; 8(8):1061-1067.

41. Bottaci L, Drew PJ, Hartley JE et al. Artificial neural networks applied to outcome prediction for colorectal cancer patients in separate institutions. Lancet 1997; 350(9076):469-472.

42. Graf W, Bergstrom R, Pahlman L, Glimelius B. Appraisal of a model for prediction of prognosis in advanced colorectal cancer. Eur J Cancer 1994; 30A(4):453-457.

43. Weiser MR, Landmann RG, Kattan MW et al. Individualized prediction of colon cancer recurrence using a nomogram. J Clin Oncol 2008; 26(3):380-385.

44. Kattan MW, Gonen M, Jarnagin WR et al. A nomogram for predicting disease-specific survival after hepatic resection for metastatic colorectal cancer. Ann Surg 2008; 247(2):282-287.

45. Blanchon F, Grivaux M, Asselain B et al. 4-year mortality in patients with non-small-cell lung cancer: development and validation of a prognostic index. Lancet Oncol 2006; 7(10):829-836.

46. Tibaldi C, Vasile E, Bernardini I, Orlandini C, Andreuccetti M, Falcone A. Baseline elevated leukocyte count in peripheral blood is associated with poor survival in patients with advanced non-small cell lung cancer: a prognostic model. J Cancer Res Clin Oncol 2008; 134(10):1143-1149.

47. Birim O, Kappetein AP, Waleboer M et al. Long-term survival after non-small cell lung cancer surgery: development and validation of a prognostic model with a preoperative and postoperative mode. J Thorac Cardiovasc Surg 2006; 132(3):491-498.

48. Moussa AS, Kattan MW, Berglund R, Yu C, Fareed K, Jones JS. A nomogram for predicting upgrading in patients with low- and intermediate-grade prostate cancer in the era of extended prostate sampling. BJU Int 2009.

49. Eastham JA, Scardino PT, Kattan MW. Predicting an optimal outcome after radical prostatectomy: the trifecta nomogram. J Urol 2008; 179(6):2207-2210.

50. Kattan MW, Wheeler TM, Scardino PT. Postoperative nomogram for disease recurrence after radical prostatectomy for prostate cancer. J Clin Oncol 1999; 17(5):1499-1507.

51. Smaletz O, Scher HI, Small EJ et al. Nomogram for overall survival of patients with progressive metastatic prostate cancer after castration. J Clin Oncol 2002; 20(19):3972-3982.

52. Escudier B, Choueiri TK, Oudard S et al. Prognostic factors of metastatic renal cell carcinoma after failure of immunotherapy: new paradigm from a large phase III trial with shark cartilage extract AE 941. J Urol 2007; 178(5):1901-1905.

53. Choueiri TK, Rini B, Garcia JA et al. Prognostic factors associated with long-term survival in previously untreated metastatic renal cell carcinoma. Ann Oncol 2007; 18(2):249-255.

54. Bochner BH, Kattan MW, Vora KC. Postoperative nomogram predicting risk of recurrence after radical cystectomy for bladder cancer. J Clin Oncol 2006; 24(24):3967-3972.

55. Lane BR, Babineau D, Kattan MW et al. A preoperative prognostic nomogram for solid enhancing renal tumors 7 cm or less amenable to partial nephrectomy. J Urol 2007; 178(2):429-434.

56. Tricot G, Spencer T, Sawyer J et al. Predicting long-term (> or = 5 years) event-free survival in multiple myeloma patients following planned tandem autotransplants. Br J Haematol 2002; 116(1):211-217.

57. Grignani G, Gobbi PG, Formisano R et al. A prognostic index for multiple myeloma. Br J Cancer 1996; 73(9):1101-1107.

58. Kaneko M, Kanda Y, Oshima K et al. Simple prognostic model for patients with multiple myeloma: a single-center study in Japan. Ann Hematol 2002; 81(1):33-36.

59. Hannisdal E, Kildahl-Andersen O, Grottum KA, Lamvik J. Prognostic factors in multiple myeloma in a population-based trial. Eur J Haematol 1990; 45(4):198-202.

60. Maucort-Boulch D, Djeridane M, Roy P, Riche B, Colonna P, Andrieu JM. Predictive and discriminating three-risk-group prognostic scoring system for staging Hodgkin lymphomas. Cancer 2007; 109(2):256-264.

61. Schot BW, Zijlstra JM, Sluiter WJ et al. Early FDG-PET assessment in combination with clinical risk scores determines prognosis in recurring lymphoma. Blood 2007; 109(2):486-491.

62. Baatenburg de Jong RJ, Hermans J, Molenaar J, Briaire JJ, le CS. Prediction of survival in patients with head and neck cancer. Head Neck 2001; 23(9):718-724.

63. Hall SF, Groome PA, Rothwell D, Dixon PF. Using the TNM system to predict survival in squamous cell carcinoma of the head and neck. Anticancer Res 1998; 18(6B):4777-4778.

64. Lee J, Lim T, Uhm JE et al. Prognostic model to predict survival following first-line chemotherapy in patients with metastatic gastric adenocarcinoma. Ann Oncol 2007; 18(5):886-891.

65. Kologlu M, Kama NA, Reis E, Doganay M, Atli M, Dolapci M. A prognostic score for gastric cancer. Am J Surg 2000; 179(6):521-526.

66. Latif AZ, Signorini D, Gregor A, Grant R, Ironside JW, Whittle IR. Application of the MRC brain tumour prognostic index to patients with malignant glioma not managed in randomised control trial. J Neurol Neurosurg Psychiatry 1998; 64(6):747-750.

67. Zhou YH, Hess KR, Liu L, Linskey ME, Yung WK. Modeling prognosis for patients with malignant astrocytic gliomas: quantifying the expression of multiple genetic markers and clinical variables. Neuro Oncol 2005; 7(4):485-494.

68. Wong SL, Kattan MW, McMasters KM, Coit DG. A nomogram that predicts the presence of sentinel node metastasis in melanoma with better discrimination than the American Joint Committee on Cancer staging system. Ann Surg Oncol 2005; 12(4):282-288.

69. Chi DS, Palayekar MJ, Sonoda Y et al. Nomogram for survival after primary surgery for bulky stage IIIC ovarian carcinoma. Gynecol Oncol 2008; 108(1):191-194.

70. Brennan MF, Kattan MW, Klimstra D, Conlon K. Prognostic nomogram for patients undergoing resection for adenocarcinoma of the pancreas. Ann Surg 2004; 240(2):293-298.

71. Kattan MW, Ficarra V, Artibani W et al. Nomogram predictive of cancer specific survival in patients undergoing partial or total amputation for squamous cell carcinoma of the penis. J Urol 2006; 175(6):2103-2108.

72. Kattan MW, Leung DH, Brennan MF. Postoperative nomogram for 12-year sarcoma-specific death. J Clin Oncol 2002; 20(3):791-796.

73. Gross ND, Patel SG, Carvalho AL et al. Nomogram for deciding adjuvant treatment after surgery for oral cavity squamous cell carcinoma. Head Neck 2008; 30(10):1352-1360.

74. Wells BJ, Jain A, Arrigain S, Yu C, Rosenkrans WA, Jr., Kattan MW. Predicting 6-year mortality risk in patients with type 2 diabetes. Diabetes Care 2008; 31(12):2301-2306.

75. Computer modeling of diabetes and its complications: a report on the Fourth Mount Hood Challenge Meeting. Diabetes Care 2007; 30(6):1638-1646.

76. Stevens RJ, Kothari V, Adler AI, Stratton IM. The UKPDS risk engine: a model for the risk of coronary heart disease in Type II diabetes (UKPDS 56). Clin Sci (Lond) 2001; 101(6):671-679.

77. Kothari V, Stevens RJ, Adler AI et al. UKPDS 60: risk of stroke in type 2 diabetes estimated by the UK Prospective Diabetes Study risk engine. Stroke 2002; 33(7):1776-1781.

78. Nguyen ND, Frost SA, Center JR, Eisman JA, Nguyen TV. Development of a nomogram for individualizing hip fracture risk in men and women. Osteoporos Int 2007; 18(8):1109-1117.

79. Kanis JA, Oden A, Johnell O et al. The use of clinical risk factors enhances the performance of BMD in the prediction of hip and osteoporotic fractures in men and women. Osteoporos Int 2007; 18(8):1033-1046.

80. Moreno RP, Metnitz PG, Almeida E et al. SAPS 3--From evaluation of the patient to evaluation of the intensive care unit. Part 2: Development of a prognostic model for hospital mortality at ICU admission. Intensive Care Med 2005; 31(10):1345-1355.

81. Zimmerman JE, Wagner DP, Draper EA, Wright L, Alzola C, Knaus WA. Evaluation of acute physiology and chronic health evaluation III predictions of hospital mortality in an independent database. Crit Care Med 1998; 26(8):1317-1326.

82. Zimmerman JE, Kramer AA, McNair DS, Malila FM. Acute Physiology and Chronic Health Evaluation (APACHE) IV: hospital mortality assessment for today's critically ill patients. Crit Care Med 2006; 34(5):1297-1310.

83. Higgins TL, Teres D, Copes WS, Nathanson BH, Stark M, Kramer AA. Assessing contemporary intensive care unit outcome: an updated Mortality Probability Admission Model (MPM0-III). Crit Care Med 2007; 35(3):827-835.

84. Teno JM, Harrell FE, Jr., Knaus W et al. Prediction of survival for older hospitalized patients: the HELP survival model. Hospitalized Elderly Longitudinal Project. J Am Geriatr Soc 2000; 48(5 Suppl):S16-S24.

85. Briggs A, Spencer M, Wang H, Mannino D, Sin DD. Development and validation of a prognostic index for health outcomes in chronic obstructive pulmonary disease. Arch Intern Med 2008; 168(1):71-79.

86. Celli BR, Cote CG, Marin JM et al. The body-mass index, airflow obstruction, dyspnea, and exercise capacity index in chronic obstructive pulmonary disease. N Engl J Med 2004; 350(10):1005-1012.

87. Puhan MA, Garcia-Aymerich J, Frey M et al. Expansion of the prognostic assessment of patients with chronic obstructive pulmonary disease: the updated BODE index and the ADO index. Lancet 2009; 374(9691):704-711.

88. Bauer TT, Ewig S, Marre R, Suttorp N, Welte T. CRB-65 predicts death from community-acquired pneumonia. J Intern Med 2006; 260(1):93-101.

89. Fine MJ, Auble TE, Yealy DM et al. A prediction rule to identify low-risk patients with community-acquired pneumonia. N Engl J Med 1997; 336(4):243-250.

90. Aujesky D, Auble TE, Yealy DM et al. Prospective comparison of three validated prediction rules for prognosis in community-acquired pneumonia. Am J Med 2005; 118(4):384-392.

91. Slotman GJ. Prospectively validated prediction of organ failure and hypotension in patients with septic shock: the Systemic Mediator Associated Response Test (SMART). Shock 2000; 14(2):101-106.

92. Altmann A, Daumer M, Beerenwinkel N et al. Predicting the response to combination antiretroviral therapy: retrospective validation of geno2pheno-THEO on a large clinical database. J Infect Dis 2009; 199(7):999-1006.

93. Tong MJ, Blatt LM, Tong LT, Sayadzadeh K, Conrad A. Long-term retreatment in chronic hepatitis C patients who were non-responders to an initial course of interferon-alpha 2b. J Viral Hepat 1998; 5(5):323-331.

94. Trapero-Marugan M, Marin M, Pivel JP et al. Predictive graphical model, network-based medical tool for the prognosis of chronic hepatitis C patients treated with peg-interferon plus ribavirin. Aliment Pharmacol Ther 2008; 28(4):468-474.

95. Trapero-Marugan M, Marin M, Pivel JP et al. Predictive graphical model, network-based medical tool for the prognosis of chronic hepatitis C patients treated with peg-interferon plus ribavirin. Aliment Pharmacol Ther 2008; 28(4):468-474.
